# High quality reference genomes for toxigenic and non-toxigenic *Vibrio cholerae* serogroup O139

**DOI:** 10.1038/s41598-019-41883-x

**Published:** 2019-04-10

**Authors:** Matthew J. Dorman, Daryl Domman, Muhammad Ikhtear Uddin, Salma Sharmin, Mokibul Hassan Afrad, Yasmin Ara Begum, Firdausi Qadri, Nicholas R. Thomson

**Affiliations:** 1 Wellcome Sanger Institute, Wellcome Genome Campus, Hinxton, Cambridgeshire CB10 1SA United Kingdom; 2 Infectious Diseases Division, International Centre for Diarrhoeal Disease Research, Dhaka, Bangladesh; 3 London School of Hygiene and Tropical Medicine, Keppel Street, London, WC1E 7HT United Kingdom

## Abstract

Toxigenic *Vibrio cholerae* of the O139 serogroup have been responsible for several large cholera epidemics in South Asia, and continue to be of clinical and historical significance today. This serogroup was initially feared to represent a new, emerging *V*. *cholerae* clone that would lead to an eighth cholera pandemic. However, these concerns were ultimately unfounded. The majority of clinically relevant *V*. *cholerae* O139 isolates are closely related to serogroup O1, biotype El Tor *V*. *cholerae*, and comprise a single sublineage of the seventh pandemic El Tor lineage. Although related, these *V*. *cholerae* serogroups differ in several fundamental ways, in terms of their O-antigen, capsulation phenotype, and the genomic islands found on their chromosomes. Here, we present four complete, high-quality genomes for *V*. *cholerae* O139, obtained using long-read sequencing. Three of these sequences are from toxigenic *V*. *cholerae*, and one is from a bacterium which, although classified serologically as *V*. *cholerae* O139, lacks the CTXφ bacteriophage and the ability to produce cholera toxin. We highlight fundamental genomic differences between these isolates, the *V*. *cholerae* O1 reference strain N16961, and the prototypical O139 strain MO10. These sequences are an important resource for the scientific community, and will improve greatly our ability to perform genomic analyses of non-O1 *V*. *cholerae* in the future. These genomes also offer new insights into the biology of a *V*. *cholerae* serogroup that, from a genomic perspective, is poorly understood.

## Introduction

*Vibrio cholerae* is the aetiological agent of cholera, an acute, life-threatening diarrhoea which has spread worldwide in seven pandemics since the nineteenth century. *V*. *cholerae* is typically sub-classified into serogroups on the basis of its somatic O-antigen. Despite there being over 200 serogroups of *V*. *cholerae*^1,2^, only serogroup O1 has caused large scale epidemics historically^3^. Previous cholera pandemics have been caused by the classical biotype of *V*. *cholerae* O1, whereas the ongoing seventh pandemic, which began in the 1960s, is caused by the El Tor biotype of *V*. *cholerae* O1^3,4^. Non-O1 serogroups of *V*. *cholerae* do not appear to cause pandemics, though they may cause outbreaks of disease. This is exemplified by an outbreak in Sudan in 1968, caused by *V*. *cholerae* O37, which was subsequently found to be genetically related to pandemic *V*. *cholerae* O1^5–8^.

In 1992, a *V*. *cholerae* clone of serogroup O139 caused a large cholera epidemic which spread rapidly across Bangladesh and India^9,10^. Due to the geographic location of the epidemic, this clone was given the name *Vibrio cholerae* O139 Bengal^10^ (dubbed *V*. *cholerae* O139 hereafter). *V*. *cholerae* O139 caused substantial numbers of cholera cases in Southeast Asia in the early 1990s, and was anticipated to emerge as the aetiological agent of an eighth cholera pandemic^11–13^. However, rather than causing a pandemic, *V*. *cholerae* O139 was associated only with a low-level incidence of cholera cases after the initial 1992–93 epidemic, until a second large outbreak occurred in Bangladesh in the Spring of 2002^14^. The re-emergence of this serogroup renewed fear that an eighth pandemic of cholera was beginning, driven by *V*. *cholerae* O139^15^. Once again, *V*. *cholerae* O139 did not proceed to cause a cholera pandemic, and although it no longer appears to be causing epidemic cholera, this serogroup has continued to be isolated since 2002. Recently, non-toxigenic *V*. *cholerae* O139 have been isolated in Thailand^16^. Toxigenic *V*. *cholerae* O139 have been isolated in China as recently as 2013^17^, and continue to be isolated in Bangladesh. Six *V*. *cholerae* O139 strains were isolated in Bangladesh between 2013 and 2014^18^ (four included in this analysis), and three more strains were isolated between 2015 and 2017.

Despite the low incidence of cholera caused by *V*. *cholerae* O139, this serogroup continues to be important to both the research and public health communities, not least because the disease caused by *V*. *cholerae* O139 is clinically indistinguishable from that caused by *V*. *cholerae* O1^10^. Accordingly, *V*. *cholerae* O139 continues to be the subject of surveillance in Southeast Asia and is included in cholera vaccine formulations^19–21^. Early genetic and biochemical studies demonstrated that O139 strains were closely related to O1 seventh pandemic El Tor strains, and it was suggested that *V*. *cholerae* O139 had arisen from an O1 El Tor ancestor^9,22–24^. This was subsequently confirmed using whole-genome sequencing, which showed that toxigenic *V*. *cholerae* O139 formed a discrete sub-lineage within the seventh pandemic El Tor (7PET) lineage^25,26^.

Although the clinical diseases caused by *V*. *cholerae* O1 and O139 are indistinguishable, there are notable differences between *V*. *cholerae* O139 and *V*. *cholerae* O1 in addition to their serogroup. For instance, *V*. *cholerae* O139 expresses a polysaccharide capsule, which 7PET *V*. *cholerae* O1 isolates lack^27^. The capsule is encoded by genes not found in other 7PET *V*. *cholerae* O1 genomes, which are located adjacent to the locus encoding lipopolysaccharide (LPS) biosynthesis genes in *V*. *cholerae* O139^15,28–32^. It has also been reported that the complement of genomic islands found in the genome of MO10, a *V*. *cholerae* O139 strain, differs from that found in 7PET *V*. *cholerae* O1^25^. The MO10 genome sequence is currently used to represent *V*. *cholerae* O139 in comparative genomic analyses, but its genome sequence is incomplete and comprises 84 contigs (assembly accession number GCA_000152425.1).

*V*. *cholerae* O139 caused 93% of laboratory-confirmed cholera cases in China in the early 2000s^19^. Such data demonstrate why *V*. *cholerae* O139 has twice been feared to be responsible for an eighth cholera pandemic. Despite the clinical importance of this serogroup which continues to be isolated today, a closed reference genome for *V*. *cholerae* O139 has not yet been published. Here, we report the first high-quality, closed reference genome sequences for this *V*. *cholerae* serogroup. We have used long-read sequencing to obtain the complete genome sequences of four recent *V*. *cholerae* O139 isolates from Bangladesh^18^. Three of these are toxigenic members of the 7PET lineage, two of which were isolated from asymptomatic patients from within a household where there had been a confirmed cholera case. The fourth isolate was acquired from a patient suffering from diarrhoea, vomiting and dehydration (see ref.^18^ for full details of the clinical history surrounding these four isolates), but is a non-toxigenic *V*. *cholerae* O139 variant that is not part of the 7PET lineage, and does not harbour the CTXφ bacteriophage^18^. This non-7PET genome offers an opportunity to study the genetic factors that enable non-toxigenic *V*. *cholerae* to cause diarrhoea in patients.

## Results

### Structure of CTXφ tandem arrays in *V. cholerae* O139

Using long-read sequencing read data, we generated single, contiguous genome sequences for the two chromosomes of each isolate sequenced in this study. Use of the corresponding short-read data for each isolate to correct these assemblies did not improve the assemblies. None of these isolates contained a third replicon, as has been reported elsewhere in other *V*. *cholerae*^33^. In order to estimate genetic distance, we mapped each sample’s corresponding short-read data^18^ to the 7PET reference genome N16961 and called single nucleotide variants (SNVs) between the mapped reads for these isolates and N16961. These SNV data are provided in Table 1, together with summary statistics for these closed assemblies. Since the three toxigenic samples were found to have near-identical genomes, varying in size by 4 bases at most, and differing by fewer than five SNVs between one another (SNVs were determined relative to N16961), 48853_H01 was selected as an exemplar sequence for further analyses.

**Table 1:** Summary of the *V*. *cholerae* O139 genome assemblies generated in this study.

Internal sequence ID (PacBio)	Sample Name	CTXφ present?	Accession (PacBio reads)	Accession (closed chromosomal assembly)	Accession (Illumina reads)	Genome Size (bp)	Average coverage of *de novo* assembly with long reads (X)	Coverage of N16961 (%)	Number of SNVs relative to N16961
48853_F01	MP_070116	No	ERR1716489	LT992490-LT992491	ERR568405	4123525	165.76	58.5	122865
48853_G01	P_0684000	Yes	ERR1716490	LT992486-LT992487	ERR568406	4092641	170.56	97	271
48853_H01	ICVB_2236_02	Yes	ERR1716491	LT992488-LT992489	ERR568407	4092645	147.29	97	270
48853_A02	SMIC_67_01	Yes	ERR1716492	LT992492-LT992493	ERR568408	4092644	165.65	97	274

The accession numbers for both the long-read sequences and assemblies generated in this study, and the original short reads used for assembly polishing, SNV calling, and phylogenetic analyses (see Methods) are reported. The SNV counts reported do not account for the removal of recombinogenic sequences, since the non-toxigenic isolate was not included in the recombination analysis. Average coverage values taken from *de novo* HGAP assemblies.

**Figure 1:**
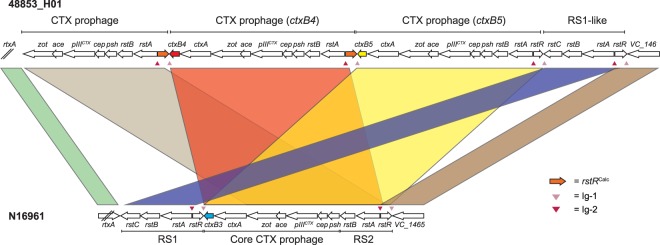
Tandem copies of the CTXφ bacteriophage in *V*. *cholerae* O139. An illustration of the genomic organisation of three tandem copies of CTXφ in the toxigenic *V*. *cholerae* O139 samples in this study. Two copies of the *ctxAB* operon are present in these genomes, which harbour different *ctxB* alleles to one another and to that of N16961. Exemplar data from the assembly for 48853_H01 are presented. Loci are not to scale. Figure annotation based on ref.^36^.

The CTXφ bacteriophage integrates into the *V*. *cholerae* chromosome in a XerCD-dependent manner, by recombination between the CTXφ *attP* site and bacterial *attB* site, which produces hybrid *attL* and *attR* sequences^34,35^. This occurs after the replicative, circular form of the viral genome forms after infection of the cell, and this integration usually involves tandem integrations of CTXφ into the genome^36^. CTXφ replication is achieved by the production of ssDNA from chromosomal tandem arrays of CTXφ in a manner dependent on the CTXφ-encoded RstA protein, where RstA nicks the CTXφ replication origin located in the intergenic region Ig-1^36,37^. The exposed 3′ site permits synthesis of CTXφ DNA up until the second, tandem CTXφ replication origin is encountered, which is also a substrate for RstA cleavage, creating a free CTXφ genome^36,37^. We observed two additional CTXφ bacteriophage sequences in tandem, relative to that found in the N16961 reference genome, located between *VC_1450* and *VC_1467* in the larger chromosome in the three toxigenic isolates (Fig. 1). A partial third repeat of CTXφ was evident, comprising the genes between and including *zot* and *rstA*, and an *rstR* open reading frame corresponding to *rstR*^*Calc*^ ^38^ (Fig. 1). We identified a complete *attL* sequence adjacent to the *VC_1465* locus in 48853_H01^35^. The phage sequence in the *attR* site adjacent to *rtxA* is not identical to that reported by Huber and Waldor^35^, although the *attR* sequence does contain both the central recombination identity sequence and the residual bacterial *attB* sequence.

Although tandem repeats of CTXφ genes in *V*. *cholerae* O139 have been reported previously^15,38,39^, difficulty in assembling these repetitive regions with short-read sequencing data meant that these repeats were not identified in our original sequencing of these isolates^18^. We mapped the short-read data to the long-read assemblies for each of these genomes, and to the N16961 reference, to confirm that short reads mapped to both the *ctxB4* and *ctxB5* variant CTXφ regions, and that when mapped to N16961, the coverage of the CTXφ region was approximately double that of the surrounding chromosome (Supplementary Fig. S1). Manual inspection of these mapping data showed that reads from both *ctxB* alleles mapped to the N16961 *ctxB* locus.

We noted that the two *ctxB* genes in these genomes are of different alleles (Fig. 1). The *ctxB* gene closest to *rtxA* in these assemblies was a *ctxB4* allele, and the second *ctxB* was a *ctxB5* allele. Both of these are *ctxB* alleles that have been found in *V*. *cholerae* O139 strains previously^40,41^. The presence of more than one *ctxB* allele in the same *V*. *cholerae* genome has not been reported previously to our knowledge, though it has been reported that *V*. *cholerae* O139 can harbour more than one type of CTXφ phage simultaneously^15,38,42^.

### Genomic islands and antimicrobial resistance genes in *V. cholerae* O139

**Figure 2:**
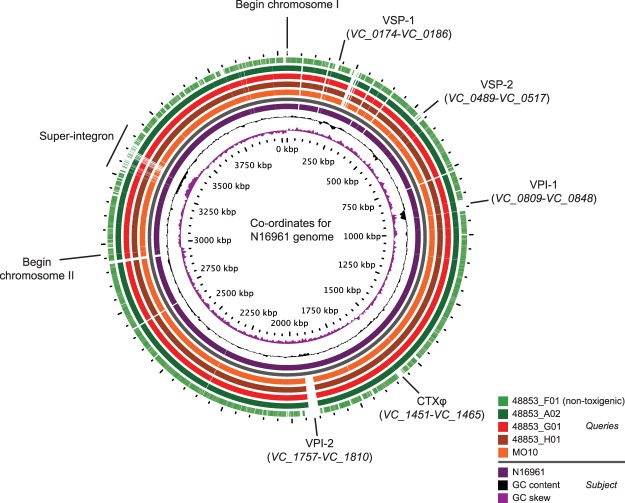
BLAST atlas comparing the genomes used in this study and MO10 to the N16961 reference genome. The presence of VPI-1, VSP-1, and VSP-2 in the three toxigenic *V*. *cholerae* O139 assemblies, as well as the truncation of VPI-2, is indicated. The non-toxigenic strain did not harbour VSP-1, VSP-2, or VPI-2 (indicated), although a region homologous to part of VPI-1 (*VC_0809* to *VC_0816*) was detected on the larger chromosome. The sequences of both *V*. *cholerae* chromosomes were concatenated to generate this figure; the boundary between the chromosomes is denoted.

Having observed these unusual CTXφ configurations, we scanned the four assemblies for the presence and absence of the genomic islands that are associated with pandemic *V*. *cholerae*: VSP-1, VSP-2, VPI-1, VPI-2, and the drug resistance genetic element SXT^25^. We identified VPI-1 and VSP-2 islands in all three of the toxigenic *V*. *cholerae* O139 isolates, and we confirmed that VPI-2 is severely truncated to the point of absence, as described previously for MO10^25,43^ (Fig. 2). We identified a genomic island integrated into the *VC_0659* locus (encoding peptide chain release factor 3) in each of the three toxigenic *V*. *cholerae* O139 assemblies, identical to SXT, which is also known as ICE*Vch*Ind4^44^. SXT is integrated into the same locus as it is in MO10. We also identified an insertion into *VC_0659* in the non-toxigenic genome assembly, which was 64% identical to ICE*Vch*Ind4 (Table 2). All of these observations agree with data from Chun *et al*.^25^ on the distribution of genomic islands in the *V*. *cholerae* O139 MO10 genome.

**Table 2:** Presence and absence of selected genomic islands in *V*. *cholerae* O139 genome assemblies.

Sample Name	VSP-1 (*VC_0174*-*VC_0186*)	VSP-2 (*VC_0489*-*VC_0517*)	VPI-1 (*VC_0809*-*VC_0848*)	VPI-2 (*VC_1757*-*VC_1810*)	CTXφ (*VC_1451*-*VC_1465*)	SXT (*VC_0659* insertion)
48853_F01	Absent	Absent	Partially present (*VC_0809-VC_0816*; deletion of *VC_0817-VC_0848*)	Absent	Absent	64% match to ICE*Vch*Ind4
48853_G01	Present, and duplication of *VC_0175*–*0186* on chr2	Present	Present	Deletion of *VC_1761*–*1787*	Present, in more than one copy	100% match to ICE*Vch*Ind4
48853_H01	Present, and duplication of *VC_0175*–*0186* on chr2	Present	Present	Deletion of *VC_1761*–*1787*	Present, in more than one copy	100% match to ICE*Vch*Ind4
48853_A02	Present, and duplication of *VC_0175*–*0186* on chr2	Present	Present	Deletion of *VC_1761*–*1787*	Present, in more than one copy	100% match to ICE*Vch*Ind4

Similarity percentages were obtained by comparing SXT element sequences to that of ICE*Vch*Ind4 using BLASTn. chr2 = chromosome 2.

In the three toxigenic *V*. *cholerae* O139 isolates, we detected the VSP-1 element integrated on the larger chromosome between genes *VC_0173* and *VC_0187*, as found in N16961^25^ (Fig. 2). However, we also observed a sequence of DNA on the smaller chromosome of each of the toxigenic isolates, integrated between *VC_A0695* and *VC_A0696*, that was 99% identical to VSP-1 (*VC_0175* to *VC_0186*; Supplementary Fig. S2). This suggested that a second copy of the VSP-1 element was present on the second chromosome in each of these genomes. We mapped the previously-published Illumina reads for these genomes^18^ to the N16961 reference genome and plotted the read depth for VSP-1 relative to the surrounding genome (Supplementary Fig. S2), which further supported the conclusion that these genomes contain a second copy of VSP-1.

This VSP-1 duplication was detected in each of the three toxigenic *V*. *cholerae* O139 genome assemblies (summarised in Table 2). It is known that VSP-1 is capable of excising from the larger *V*. *cholerae* chromosome^45^, and it has been previously reported that the Matlab variant *V*. *cholerae* O1 strain MJ-1236 harbours a second copy of VSP-1 integrated between *VC_A0695* and *VC_A0696*^46^. Grim *et al*.^46^ used PCR to identify a single clinical isolate of *V*. *cholerae* O139 from Bangladesh that harboured an insertion between *VC_A0695* and *VC_A0696* resembling VSP-1, but this isolate was not described further. This phenomenon is likely to be that which we have now confirmed to be present in these three *V*. *cholerae* O139 genomes.

We also compared the genomic island complement of these four sequences with that of MO10. MO10 harbours a kappa prophage (GI-11^25^), which is absent from N16961 and also absent from the four sequences in this study (Supplementary Fig. S3). Likewise, MO10 harbours the *Vibrio* VSK prophage (GI-16^25^), which is absent from both N16961 and the O139 sequences in this study (Supplementary Fig. S3). MO10 does not appear to harbour the second VSP-1 copy on chromosome 2 which we identified in the three toxigenic isolates. The SXT variant harboured by MO10 is expanded relative to that found in these strains (Supplementary Fig. S3), and this expansion includes genes conferring resistance to the antimicrobials streptomycin (*strAB*), sulfamethoxazole (*sul2*), trimethoprim (*dfr18*), and chloramphenicol (*floR*). We scanned the assemblies for the four O139 genomes for antimicrobial resistance genes. The non-toxigenic 48853_F01 genome does not harbour any known antimicrobial resistance genes. The three toxigenic O139 genomes also do not contain any antimicrobial resistance genes, though they do harbour a *catB9* gene that is known not to confer antibiotic resistance^47^. These data are concordant with the original antimicrobial sensitivity testing of these isolates, which found that they were resistant only to nalidixic acid^18^. We confirmed that these four isolates harbour an S83I mutation in GyrA, and that 48853_F01 also contains an A171S mutation in GyrA and a S85L mutation in ParC. All of these mutations are associated with nalidixic acid resistance in *V*. *cholerae*^48^. We also scanned the assembled genomes of the four isolates for the presence of *V*. *cholerae* accessory virulence genes, to determine whether candidate virulence genes were present in the genome of the otherwise non-toxigenic *V*. *cholerae* O139 isolate^18^ (Table 3). We did not identify any virulence determinants in the 48853_F01 genome assembly other than those typically found in *V*. *cholerae*^3^.

**Table 3:** Presence and absence of accessory virulence genes in *V*. *cholerae* O139 genome assemblies.

Accessory virulence gene (N16961 locus ID or accession number)	Present in 48853_F01	Present in 48853_G01	Present in 48853_H01	Present in 48853_A02
ToxR (*VC_0984*)	Yes	Yes	Yes	Yes
Zona occludens toxin, Zot (*VC_1458*)	No	Yes	Yes	Yes
Accessory cholera enterotoxin, Ace (*VC_1459*)	No	Yes	Yes	Yes
Haemolysin, *hlyA* (*VC_A0219*)	Yes	Yes	Yes	Yes
Mannose-sensitive haemagglutinin, MSHA (*VC_0398*..*VC_0414*)	Yes	Yes	Yes	Yes
MARTX toxin, *rtxA* (*VC_1451*)	Yes	Yes	Yes	Yes
MARTX toxin accessory gene, *rtxC* (*VC_1450*)	Yes	Yes	Yes	Yes
HA/protease, *hapA* (*VC_A0865*)	Yes	Yes	Yes	Yes
Heat-stable enterotoxin NAG-ST (Accession # M85198.1)	No	No	No	No
Type III secretion system from *V*. *cholerae* AM_19226 (typically present *in lieu* of VPI-2; accession # AATY01000000)	No	No	No	No

Gene presence and absence was determined using ACT^67^ to visualise BLASTn synteny plots, and using tBLASTx to scan assemblies using the NAG-ST nucleotide sequence as a query.

### Phylogenetic analysis

**Figure 3:**
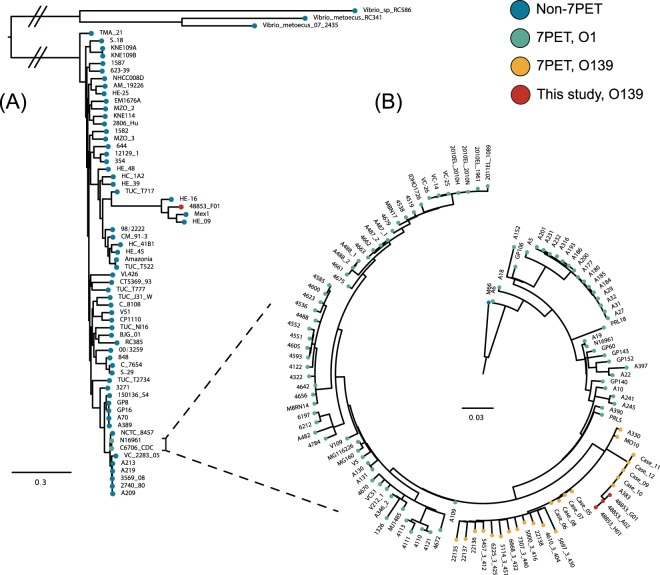
Phylogenetic analysis. (**A**) A total of 168,476 variable sites from a core-gene alignment of 2,103 genes from 65 genomes were used to generate a maximum-likelihood phylogenetic tree of the *V*. *cholerae* species, rooted on *Vibrio metoecus* and *Vibrio sp*. RC586. Two genomes that are representative of the 7PET lineage were included. The non-toxigenic isolate 48853_F01 clustered together with other non-toxigenic, non-O1/O139 *V*. *cholerae*. (**B**) A maximum-likelihood phylogeny of the 7PET lineage constructed using 1,629 non-recombinant variable sites across 117 *V*. *cholerae* genomes, rooted on M66-2. The three toxigenic samples in this study clustered together with other toxigenic O139 genome sequences, all of which form a discrete sub-lineage within 7PET. Hatch marks denote branches that were shortened artificially for illustrative purposes; an unedited tree is presented in Supplementary Fig. S5. Trees were also computed using an ascertainment bias correction model (see Methods). These are presented in Supplementary Fig. S6.

We constructed a maximum-likelihood phylogeny from an alignment of core genes from 65 diverse *V*. *cholerae* genomes, and confirmed that despite its serogroup, 48853_F01 is not a member of the 7PET O139 sublineage (Fig. 3A). We did find that the three toxigenic genomes were members of 7PET, and we used the previously-published short-read data for these genomes to place these isolates into phylogenetic context with 114 other *V*. *cholerae*, including 23 O139 genome sequences^16,18,26^ (117 genomes in total; Supplementary Table S1). We found that the three toxigenic isolates in this study clustered together with other 7PET *V*. *cholerae* O139 sequences from Bangladesh and India from 1992 to 2002 (Fig. 3B). The closest relatives of these three strains, which were isolated in 2013 and 2014, were isolated from Bangladesh in 2002 (A383, Case_09–12). All of these were also closely related to *V*. *cholerae* O139 samples from 1992–1995, including a recently-sequenced collection of *V*. *cholerae* O139 from Thailand^16^. These results recapitulate and reinforce previously-published data^18^, adding to the utility of these genomes as reference sequences for toxigenic *V*. *cholerae* O139. Moreover, there are no complete genome assemblies for non-toxigenic *V*. *cholerae* O139. Given that this isolate is clearly distinct from the 7PET O139 sublineage, we anticipate that this genome sequence will enable comparative genomic studies of *V*. *cholerae* other than 7PET isolates.

The previous report of these genome sequences, obtained using short-read technology alone, examined the structure of the capsule and lipopolysaccharide (LPS) synthesis loci. These analyses were performed using incompletely-assembled genome sequences^18^. We used the closed sequences obtained in this project to compare these loci across the strains in this study to N16961 and MO10 (Supplementary Fig. S4). We confirmed that the three toxigenic strains contain O139 LPS operons that strongly resemble that found in MO10 (Supplementary Fig. S4). The equivalent region in the non-toxigenic isolate 48853_F01 is less similar, although this strain exhibits a strong O139-positive phenotype using the rapid dipstick assay and slide agglutination tests^18^. In our phylogenetic analyses, we noted that 48853_F01 clustered with two Haitian non-O1 *V*. *cholerae* isolates from 2010 and a Mexican isolate from 1991 for which there are no serotype data (Fig. 3A; Supplementary Table S1). We found that, although these three non-O1 *V*. *cholerae* share capsule biosynthesis genes with 48853_F01, they do not harbour the same LPS operon (Supplementary Fig. S4). These Haitian and Mexican isolates therefore are unlikely to be *V*. *cholerae* of serogroup O139.

## Discussion

It is essential to have complete and accurate reference sequences to perform bacterial genomic analysis. Although several studies have provided closed *V*. *cholerae* sequences^49–53^, none to date have provided reference sequences for *V*. *cholerae* O139. The sequences in this study will serve as an important community resource in future studies of *V*. *cholerae* genomics and phylogenetics. For example, access to the closed sequences of the O139 LPS and capsule biosynthesis operons from these four strains means that it should be possible to serotype *V*. *cholerae* O139 sequences *in silico*. The fact that the LPS biosynthesis loci in these toxigenic and non-toxigenic strains are similar but not identical (Supplementary Fig. S4), and that the non-toxigenic strain is distantly related to the toxigenic *V*. *cholerae* O139 in this study (Fig. 3), suggests that there may be more than one genetic configuration that confers an O139 serogroup phenotype. In the absence of candidate virulence genes, putative or otherwise, we also cannot exclude the possibility that the non-toxigenic isolate was obtained from a patient who was co-infected with another toxigenic organism such as enterotoxigenic *Escherichia coli*^54–56^.

Many of the observations in this study could only be made because of the resolution offered by long-read sequencing. For example, the observation that several *ctxB* alleles can co-exist in a single genome is striking. The co-existence of several CTXφ sequences in tandem has been reported before, such as in the O395 classical reference sequence^52,57^ and in the PA1849 second-pandemic classical isolate^4^. However, in PA1849, the tandem bacteriophages are of the same *ctxB* allele, and in O395, the CTXφ array on the larger chromosome consists of one intact CTXφ and one partial prophage sequence^57^. Although these *V*. *cholerae* O139 had been sequenced previously, it had not been possible to assemble these genomes fully with the short-read Illumina technology used at the time. Consequently, in all three assemblies, CTXφ was not assembled into a single contig, and only one of the two *ctxB* genes was identifiable (our Illumina assemblies for 48853_G01 and 48853_H01 contain a *ctxB4* allele in a small contig, and 48853_A02 contains *ctxB5* in a larger contig). In future studies of *V*. *cholerae* O139, mapping sequencing reads against these reference sequences will address this problem, which will not be resolved if data are exclusively mapped to N16961 or related sequences.

Furthermore, we note that the oligonucleotides used for PCR based *ctxB* typing^40^ are 100% homologous to regions upstream and downstream of both *ctxB* loci in these three genomes. It would therefore not be possible to discriminate between *ctxB* types based on Sanger sequencing of these amplicons. This suggests that caution should be used in the interpretation of PCR-based *ctxB* typing data in the epidemiological study of cholera outbreaks, particularly if this CTXφ configuration is present in other *V*. *cholerae* lineages.

The functions of VSP-1 and VSP-2 are not fully understood, although it is well-accepted that these two genomic islands are found in *V*. *cholerae* which are members of the seventh pandemic^25^. It is known that DncV, encoded by VSP-1, represses *V*. *cholerae* chemotaxis^58^ and that repression of chemotaxis has been linked to improved intestinal colonisation by *V*. *cholerae*^59^. DncV is also known to be upregulated under conditions of gastrointestinal infection, in response to conditions that activate the ToxT transcription factor *via* the TarB small RNA which prevents the production of VPI-1-encoded VspR. It is interesting to speculate that the duplication of VSP-1 might further attenuate *V*. *cholerae* chemotaxis under colonisation conditions *via* a gene dosage effect, thereby modulating the ability of these *V*. *cholerae* O139 strains to colonise the intestine.

Here, by sequencing *V*. *cholerae* O139 using long-read technology, we have highlighted genomic features that emphasise the genetic distinctions between *V*. *cholerae* O139 and *V*. *cholerae* O1. We have identified differences between these recent strains, N16961, and MO10, the *V*. *cholerae* O139 strain used for previous comparative analyses. We have also described unusual phenomena in *V*. *cholerae* O139 genome biology – namely, the co-existence of more than one *ctxB* allele, and the cross-chromosome duplication of VSP-1. There also appears to have been genetic changes within *V*. *cholerae* O139 that has occurred since its first identification in 1992, typified by the MO10 isolate. Given that *V*. *cholerae* O139 is a member of 7PET, has several characteristics of a sublineage with the potential to cause pandemic disease, and continues to be isolated in recent years, research into this serogroup should continue. These reference sequences enable such research, and as well as providing interesting insights into the genome structure of recent *V*. *cholerae* O139, these sequences are an important resource for future genomic studies of *V*. *cholerae* as a pathogen and as a species.

## Methods

### Isolates and sequences used in this study

Four previously-described *V*. *cholerae* O139 isolates^18^ were selected for re-sequencing on the PacBio RSII platform. A set of 178 genomes in addition to these were included in comparative genome analyses (182 genomes in total; Supplementary Table S1).

### DNA isolation

Genomic DNA was prepared from 25 ml cultures of bacterial isolates grown overnight at 37 °C in LB media. Cells were harvested by centrifugation and resuspended in 2.0 ml of 25% w/v sucrose in TE buffer (10 mM Tris pH 8.0, 1 mM EDTA pH 8.0). Nuclei Lysis Solution (Promega, #A7941, 6.0 ml) was added and samples were lysed by incubation at 80 °C for five minutes. Samples were mixed with proteinase K (250 μg/ml final concentration), RNase A Solution (Promega, #A797A, 15 μg/ml final concentration), EDTA pH 8.0 (25 mM final concentration) and aqueous SDS solution (0.3% final concentration). Mixtures were incubated on ice for two hours and then at 50 °C overnight. Following enzymatic treatment, TE buffer was added to each sample (12 ml final volume). DNA was then isolated by phenol-chloroform extraction. Nucleic acids were precipitated in absolute ethanol, washed in ethanol (70% v/v), and resuspended in approximately 350 μl Tris (10 mM; pH 8.0). EDTA was omitted from the resuspension solution, to avoid interference with PacBio sequencing chemistry.

### Long-read sequencing

SMRTbell libraries were created from approximately 10 μg DNA according to the manufacturer’s protocol (15 kb library size, no size selection). Long reads were generated by sequencing on the PacBio RSII platform using polymerase version P6 and C4 sequencing chemistry. Sequence reads were assembled using HGAP v3^60^ of the SMRT analysis software v2.3.0. The fold coverage to target when picking the minimum fragment length for assembly was set to 30 and the approximate genome size was set to 3 Mbp. The HGAP assembler assembled the reads from sample 48853_G01 into three contigs. However, assembling this sample with Canu v1.1^61^ produced an assembly of two contigs, one per chromosome, and this was used for subsequent analysis. Assemblies were circularised using Circlator v1.1.3^62^ and the pre-assembled reads (also known as corrected reads). The circularised assemblies were polished using the PacBio RS_Resequencing protocol and Quiver v1 of the SMRT analysis software v2.3.0. Automated annotation was performed using Prokka v1.11^63^ and genus specific databases from RefSeq^64^. Pilon v1.19^65^ did not identify any SNVs in any of the PacBio assemblies using the corresponding short-read data – accordingly, no short-read corrections were made to these assemblies. Raw sequencing reads and the genome assemblies described in this study have been deposited into the European Nucleotide Archive (Table 1; Supplementary Table S1).

### Comparative genomics and BLAST atlas construction

The four annotated genome assemblies were compared to one another, and to the N16961 and MO10 reference genomes (see Supplementary Table S1 for accession numbers), using BLASTn^66^. These comparisons were visualised using ACT^67^ and by BLAST atlas comparison using the GView web server (https://server.gview.ca/).

### Read alignment, SNV identification, and core gene alignment

Paired-end Illumina reads from 116 7PET *V*. *cholerae* O1 and O139 samples, together with the M66-2 pre-pandemic strain (117 genomes in total; Supplementary Table S1), were mapped to the *V*. *cholerae* O1 El Tor N16961 reference genome (see Supplementary Table S1 for accession numbers) using SMALT v.0.7.4. Variable sites were identified using samtools mpileup v0.1.19, with parameters “-d 1000 –DsugBf”, and bcftools v0.1.19^68^. High quality SNVs were determined as previously described^69^, and putative recombinant regions were detected and filtered from the alignment using Gubbins^70^, to produce a final alignment of 1,629 SNVs.

Prokka-annotated assemblies for 63 non-7PET and two 7PET genomes^63,71^ were used to generate a species-level pan-genome using Roary^72^ with the following arguments: “-e–mafft -s -cd 97”. Poorly-aligned and gap-rich sites were removed from an alignment of 2,103 core gene sequences using trimAl v1.4.rev5^73^ with the “-automated1” argument. A total of 168,476 variable sites were identified in the resultant alignment using SNP-sites v2.3.2^74^.

### Phylogenetic analysis

Maximum likelihood phylogenetic trees were constructed using RAxML v8.2.8^75^ under the GTR model with the gamma distribution to model site heterogeneity (GTRGAMMA), using 500 bootstrap replicates. An alignment composed of 1,629 non-recombinant variable sites was used to generate a reference-based 7PET phylogeny. An alignment of 168,476 variable sites from 2,103 core genes was used to generate a core-gene *V*. *cholerae* species phylogeny. Trees were also computed using the GTR + ASC model in IQ-Tree v1.5.5^76^, optimised for an input containing no invariant nucleotides, and were supported by 5,000 ultrafast bootstrap approximations and approximate likelihood ratio tests^77–79^. Phylogenetic trees were visualised using FigTree v1.4.3 (http://tree.bio.ed.ac.uk/software/figtree/) and the interactive tree of life (iTOL) v3^80^.

### Identification of antimicrobial resistance genes

Genome assemblies were scanned for the presence of antimicrobial resistance genes using the ResFinder web server v2.1 (https://cge.cbs.dtu.dk/services/ResFinder/)^81^.

## Supplementary information

Supplementary material for High quality reference genomes for toxigenic and non-toxigenic <i>Vibrio cholerae</i> serogroup O139

Supplementary Table S1

## Data Availability

Sequencing reads generated during this project have been deposited into the European Nucleotide Archive (ENA; http://www.ebi.ac.uk/ena) under study accession number PRJEB14661. Assembled genome sequences have been deposited under accession numbers LT992486- LT992493. Sequence alignments, phylogenetic tree data, and other supporting information are available in Figshare (10.6084/m9.figshare.6480266).
