# Supplementary material for: High quality reference genomes for toxigenic and non-toxigenic Vibrio cholerae serogroup O139
Source: Sci Rep. 2019 Apr 10;9:5865. doi: 10.1038/s41598-019-41883-x (PMC6458141; doi:10.1038/s41598-019-41883-x)
Supplement: Supplementary file 1 — Supplementary material for High quality reference genomes for toxigenic and non-toxigenic Vibrio cholerae serogroup O139 [file 41598_2019_41883_MOESM1_ESM.pdf]

Supplementary material for

High quality reference genomes for toxigenic and non-toxigenic *Vibrio cholerae* serogroup O139.

Matthew J. Dorman, Daryl Domman, Muhammad Ikhtear Uddin, Salma Sharmin, Mokibul Hassan Afrad, Yasmin Ara Begum, Firdausi Qadri & Nicholas R. Thomson

These supplementary materials include:

Table S1. List of strains and genome sequences used in this study (attached .xls spreadsheet).

Figures S1-S6.

Supplementary references. Additional references for cited data and supplementary figures.

Additional materials that support this study are available in Figshare: https://dx.doi.org/10.6084/m9.figshare.6480266

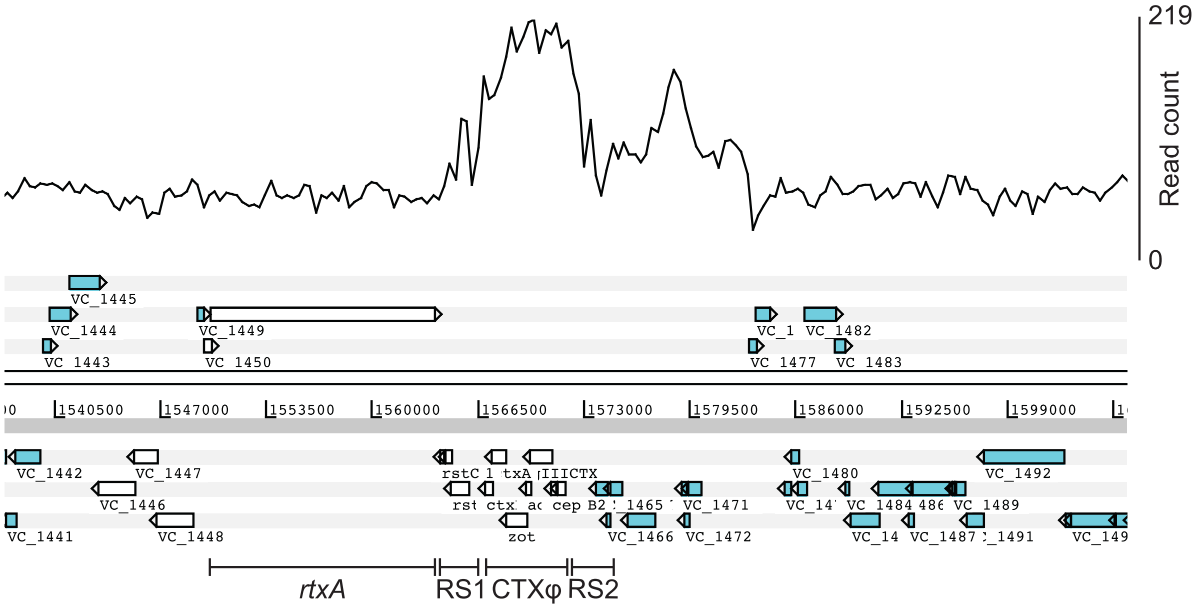

Figure S1. Confirmation of the duplication of the CTXφ bacteriophage in 48853_H01.

Short reads for 48853_H01 were mapped against the N16961 reference genome (see Methods). Mapping data were visualised using Artemis [82].

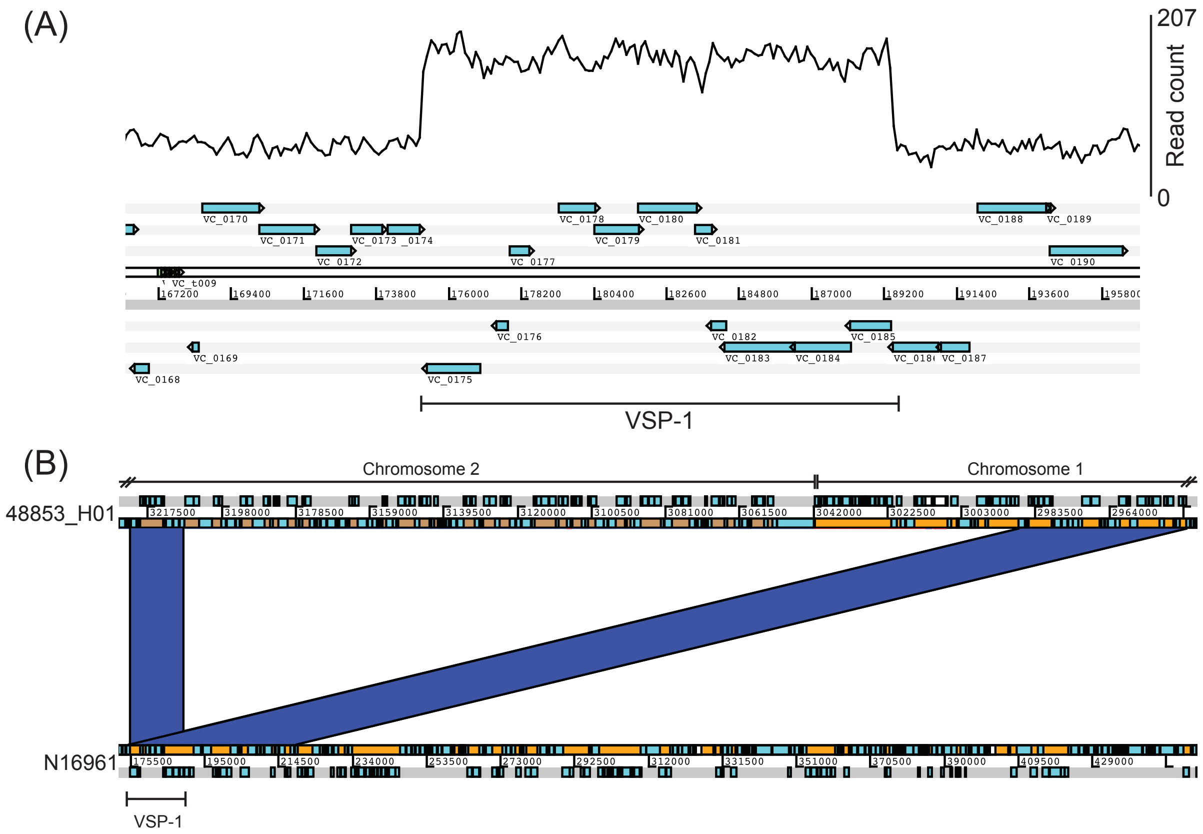

Figure S2. Confirmation of a duplication of VSP-1 in 48853_H01. (A): Short reads for 48853_H01 were mapped against the N16961 reference genome. Mapping data suggested that approximately double the number of reads mapping to VSP-1 were present relative to those mapping to the surrounding chromosome. (B): A region homologous to VSP-1 was detected on both chromosomes 1 and 2 in the 48853_H01 assembly. Data were visualised using Artemis and BamView [82,83] and ACT [67].

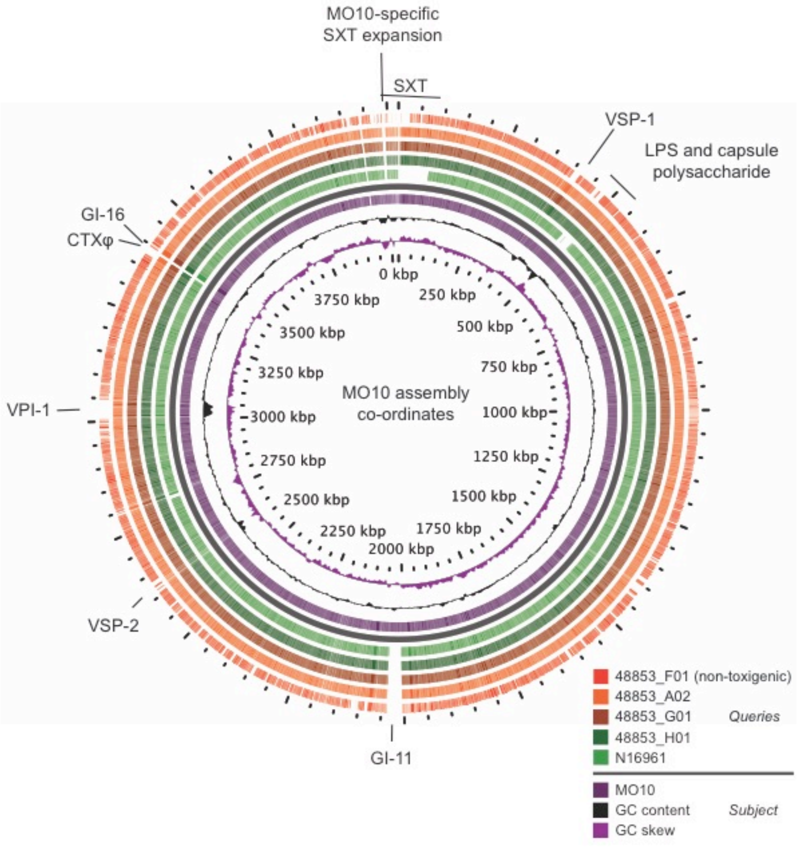

Figure S3. BLAST atlas comparing the four closed assemblies from this study, and that of N16961, to MO10. The loci harbouring GI-11, GI-16, and SXT are also indicated. VPI-2 is not present in the MO10 genome. MO10 harbours an expanded SXT variant. The O139 LPS and capsule-production genes are absent from N16961 (Supplementary Fig. S4). The sequences of both *V. cholerae* chromosomes (*i.e.*, all MO10 contigs) were concatenated to generate this figure.

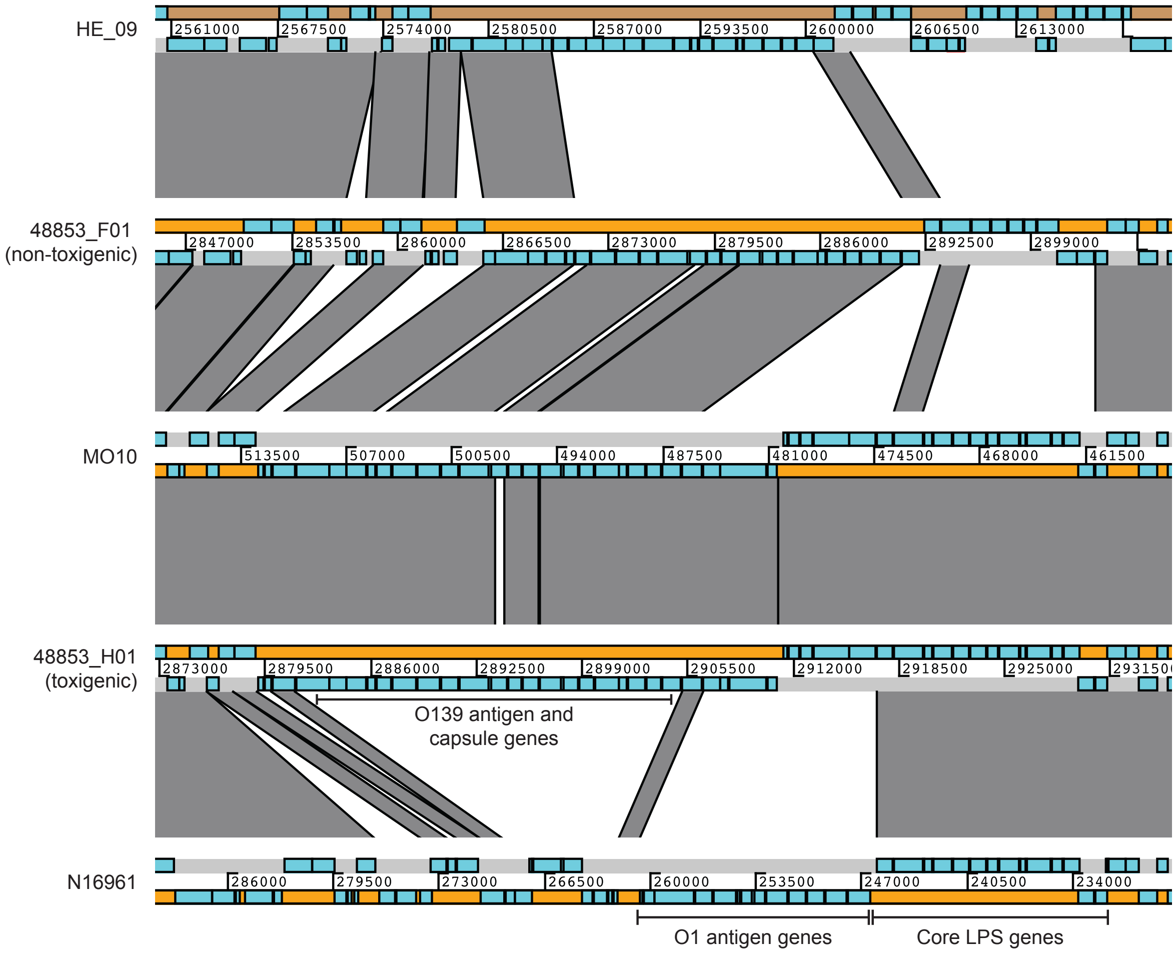

Figure S4. Comparison of the structure of capsule and lipopolysaccharide biosynthesis loci in five *V. cholerae*. The genes encoding the O1 antigen are absent in 48853_H01, replaced with those encoding the O139 antigen and polysaccharide capsule. These are common to MO10, and partially conserved in the non-toxigenic strain. Core LPS genes vary between MO10 and 48853_F01, and O-antigen genes are not shared between 48853_F01 and the three non-7PET *V. cholerae* to which it is most closely related (Fig. 3A), represented here by HE_09. Syntenic regions were visualised using ACT [67].

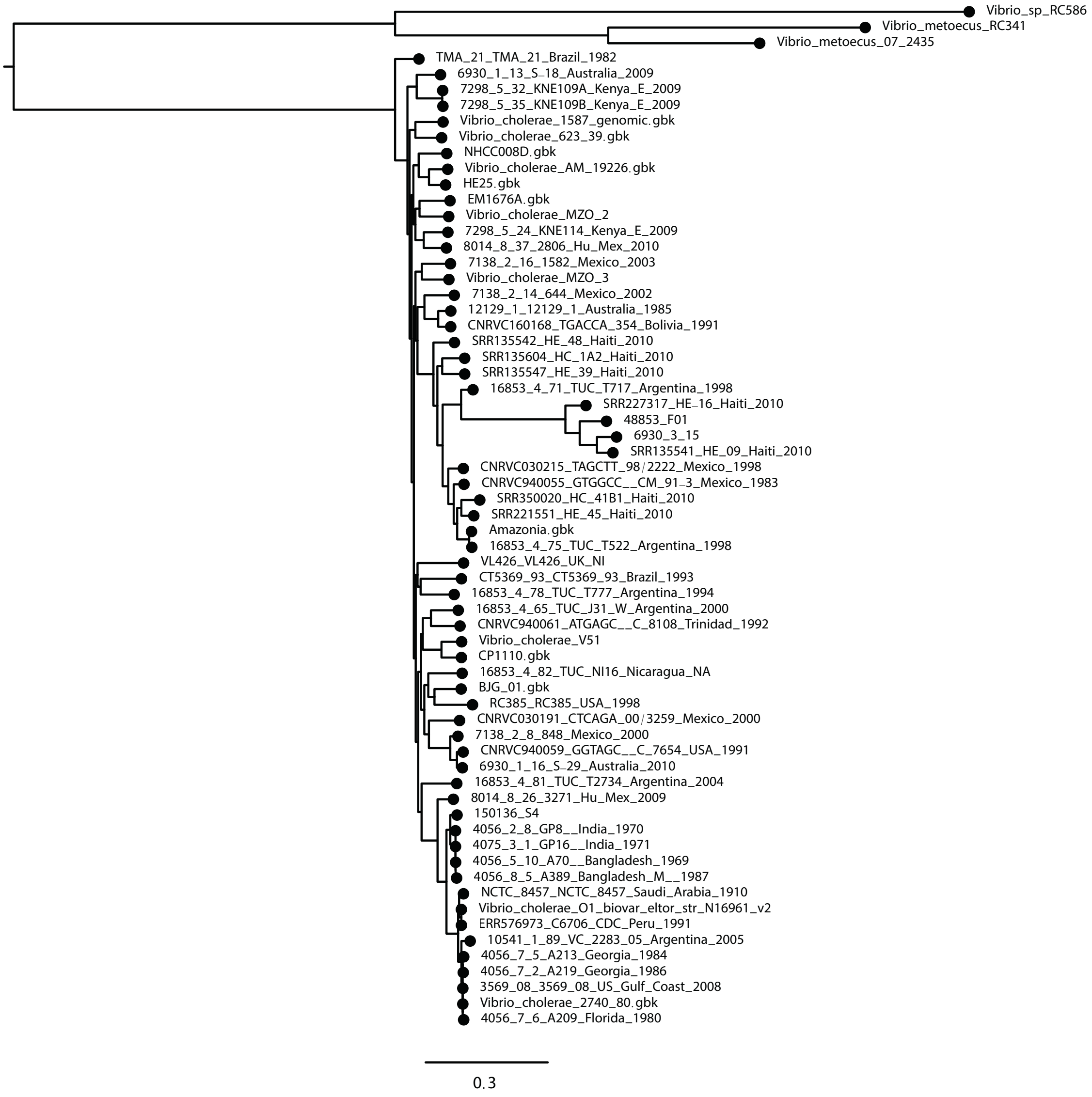

Figure S5. Unedited *V. cholerae* species phylogeny. The non-7PET phylogeny reported in Fig. 3A is presented without artificial branch shortening.

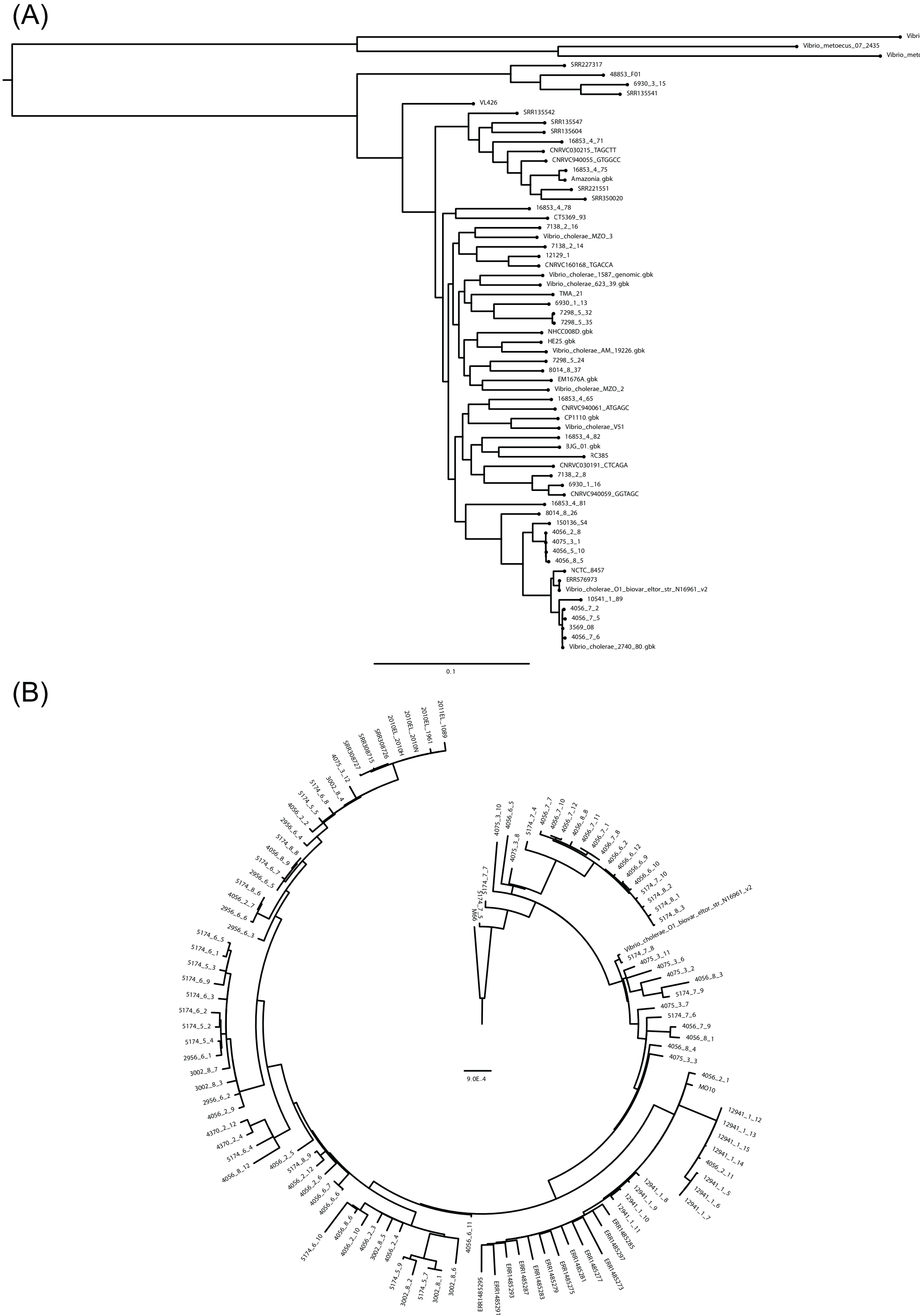

Figure S6. Phylogenetic trees computed under the GTR+ASC model. Phylogenies computed using an ascertainment bias correction model from the alignments used to generate Fig. 3A and Fig. 3B are presented (A and B, respectively).

Supplementary references. Additional references for cited data and supplementary figures.

82. Rutherford, K. *et al.* Artemis: sequence visualization and annotation. *Bioinformatics* **16,** 944–945 (2000).

83. Carver, T., Böhme, U., Otto, T. D., Parkhill, J. & Berriman, M. BamView: viewing mapped read alignment data in the context of the reference sequence. *Bioinformatics* **26,** 676–677 (2010).

84. Haley, B. J. *et al.* Comparative genomic analysis reveals evidence of two novel *Vibrio* species closely related to *V. cholerae*. *BMC Microbiol.* **10,** 154 (2010).

85. Haley, B. J. *et al.* Genomic and phenotypic characterization of *Vibrio cholerae* non-O1 isolates from a US Gulf Coast cholera outbreak. *PLoS ONE* **9,** e86264 (2014).

86. Hasan, N. A. *et al.* Genomic diversity of 2010 Haitian cholera outbreak strains. *Proc. Natl. Acad. Sci.* **109,** E2010–E2017 (2012).

87. Islam, A. *et al.* Indigenous *Vibrio cholerae* strains from a non-endemic region are pathogenic. *Open Biol.* **3,** 120181 (2013).

88. Kiiru, J. *et al.* A study on the geophylogeny of clinical and environmental *Vibrio cholerae* in Kenya. *PLoS ONE* **8,** e74829 (2013).

89. Orata, F. D. *et al.* The dynamics of genetic interactions between *Vibrio metoecus* and *Vibrio cholerae*, two close relatives co-occurring in the environment. *Genome Biol. Evol.* **7,** 2941–2954 (2015).

90. Reimer, A. R. *et al.* Comparative genomics of *Vibrio cholerae* from Haiti, Asia, and Africa. *Emerg. Infect. Dis.* **17,** 2113–2121 (2011).

91. Thompson, C. C. *et al.* Genome sequence of the human pathogen *Vibrio cholerae* Amazonia. *J. Bacteriol.* **193,** 5877–5878 (2011).

92. Hendriksen, R. S. *et al.* Population genetics of *Vibrio cholerae* from Nepal in 2010: Evidence on the origin of the Haitian outbreak. *mBio* **2,** e00157-11 (2011).

93. Didelot, X. *et al.* The role of China in the global spread of the current cholera pandemic. *PLoS Genet.* **11,** e1005072 (2015).
